# RBF neural network based backstepping terminal sliding mode MPPT control technique for PV system

**DOI:** 10.1371/journal.pone.0249705

**Published:** 2021-04-08

**Authors:** Zain Ahmad Khan, Laiq Khan, Saghir Ahmad, Sidra Mumtaz, Muhammad Jafar, Qudrat Khan

**Affiliations:** 1 Department of Electrical and Computer Engineering, COMSATS University Islamabad, Abbottabad Campus, Abbottabad, Pakistan; 2 Department of Electrical and Computer Engineering, COMSATS University Islamabad, Islamabad, Pakistan; 3 School of Electrical Engineering, National University of Computer and Emerging Sciences Islamabad, Islamabad, Pakistan; 4 Center for Advanced Studies in Telecommunication, COMSATS University Islamabad, Islamabad, Pakistan; Huazhong University of Science and Technology, CHINA

## Abstract

The energy demand in the world has increased rapidly in the last few decades. This demand is arising the need for alternative energy resources. Solar energy is the most eminent energy resource which is completely free from pollution and fuel. However, the problem occurs when it comes to efficiency under different atmospheric conditions such as varying temperature and solar irradiance. To achieve its maximum efficiency, an algorithm of maximum power point tracking (MPPT) is needed to fetch maximum power from the photovoltaic (PV) system. In this article, a nonlinear backstepping terminal sliding mode control (BTSMC) is proposed for maximum power extraction. The system is finite-time stable and its stability is validated through the Lyapunov function. A DC-DC buck-boost converter is used to deliver PV power to the load. For the proposed controller, reference voltages are generated by a radial basis function neural network (RBF NN). The proposed controller performance is tested using the MATLAB/Simulink tool. Furthermore, the controller performance is compared with the perturb and observe (P&O) MPPT algorithm, Proportional Integral Derivative (PID) controller and backstepping MPPT nonlinear controller. The results validate that the proposed controller offers better tracking and fast convergence in finite time under rapidly varying conditions of the environment.

## 1 Introduction

Energy plays a vital role in a modern-day economy. It is essential in running the machines of industrial units and factories, lightening the cities and empowering the vehicles. Due to the population growth and development of industries, the demand of energy has been immensely increased. In this regard, many types of energy resources are explored for power generation such as solar, wind, biomass, geothermal, etc. Solar energy is one of the most reliable, abundantly available, and prominent sources of energy. In 2015, Globally 55 Gigawatts (GWs) of solar energy have been added to installed capacity which is a remarkable achievement [1]. The sun gives infinite energy to the planet earth which is effortlessly available everywhere and to everyone. It is completely sustainable. Solar panels never cause any type of pollution.

PV systems can be connected with the electrical grid or load. Although the PV system’s installation cost is low, it has the problem of efficiency that varies with the atmospheric conditions [2]. The four major components of a PV system are the PV panel, DC-DC converter, MPPT controller, and electric load. The direct connection between the PV array and load is inefficacious. A buck-boost converter is used as an interface between a PV array and electric load. The duty cycle of the converter is optimally varied in a way where the PV module is operated at maximum voltage and current. This phenomenon ensures that the PV module is extracting the maximum power and operating at a maximum power point (MPP) [3]. According to one diode model of a PV system, continuously changing the environmental conditions like temperature and irradiance keep on varying the maximum voltage [2]. But the problem is to extract maximum power from the available PV panels. To fetch maximum power, PV panels must operate at MPP. MPPT is a commonly used technique that is employed to achieve maximum power from the energy source. The objective of this technique is to get optimal MPP operation in different conditions of environment [4].

Different techniques and algorithms are applied to achieve maximum power point from the PV system. Almost all MPPT techniques have to track current, *I*_*mpp*_ or voltage, *V*_*mpp*_ at maximum power point at which module of PV will deliver the maximum power. Tracking the *V*_*mpp*_ or *I*_*mpp*_ is an important process to ensure efficient utilization of a PV system.

The conventional methods are based on the hill-climbing technique, mainly including two algorithms, perturb and observe P&O MPPT algorithm and incremental conductance (IC) MPPT algorithm. P&O algorithm is the simplest technique to fetch maximum power. The basic concept behind this algorithm is to perturb the voltage or current of the PV panel and perceives any difference in the extracted power. The perturbation is presented due to changes in the duty cycle of the electronic converter. The power difference, Δ*P* is checked at various levels of voltage. The converter’s duty cycle is varied to achieve MPPT. This process of perturbation and observation takes time and repeats as well at every stage. In the end, the PV system attains MPP. The oscillations occur around the MPP. Thus, the system efficiency is reduced [5–7]. IC algorithm is another commonly used method to achieve MPP. The drawback of the P&O technique is encountered in the IC MPPT algorithm. The oscillation in MPP can be obliterated in this method of MPPT by comparing the incremental conductance of the panel, Δ*I*_*pv*_/Δ*V*_*pv*_ and instantaneous conductance of panel, *I*_*pv*_/*V*_*pv*_. MPP is attained, when the sum of Δ*I*_*pv*_/Δ*V*_*pv*_ and *I*_*pv*_/*V*_*pv*_ is zero. The implementation is simple. Its accuracy in tracking and better efficiency gives an edge over P&O algorithm [8, 9]. Thus, this technique requires some extra circuitry of control for better performance under variant conditions of environment [10].

Optimization-based algorithms like particle swarm optimization (PSO) algorithm, genetic algorithm (GA), ant colony optimization (ACO) algorithm, flower pollination algorithm (FPA), artificial bee colony (ABC), firefly algorithm (FFA), shuffled frog leaping algorithm (SFLA) and grey wolf (GW) optimization algorithm are used for achieving maximum power but these all are population-based algorithms [11–16]. Although these algorithms fix the partial shading issue, the performance of these algorithms highly depends upon the initial conditions and selected parameters. Many parameters like chromosome selection, size of the population, crossover rate, and mutation rate are needed. These requirements enhance the computational complexity and steady-state time [17].

Artificial Intelligence (AI) techniques exhibit advantages over conventional methods. They can deal with the input variables and handle system nonlinearity. There is no need for a system mathematical model. As compared with the conventional MPPT techniques, AI control techniques are more robust. Artificial neural network (ANN) and fuzzy logic controller (FLC) are types of AI techniques. FLC needs a rule base table and performs well under varying irradiance. When FLC is combined with other MPPT algorithms like P&O [18] and ANN [19] then it is highly appreciable. For designing the FLC rules, plenty of system knowledge and more training is required. That requires a huge amount of memory and processing time. Adaptive fuzzy logic controller (AFLC) is proposed to achieve MPPT which operates into two rules [20]. In the first one, the duty cycle of the electronic converter is adjusted while the controller gain is adjusted in the second rule. In [21], adaptive neuro-fuzzy inference system technique is applied to get MPPT for the PV system under different atmospheric conditions. Fuzzy based controllers are developed with expert system knowledge to produce MPPT rules. ANN method is gleaned from the behavior of the a human being. ANN has the thinking ability to decide and more knowledge is required for the training of neurons. It has three layers; input, hidden, and output. In [22], ANN based FLC for MPPT fetching is proposed. ANN and FLC based MPPT techniques results in better performance as compared to the optimization based algorithms. Demerits are implementation complexity and computational cost. These MPPT controllers need regular maintenance because the PV array electricity curve changes over time.

Many linear controllers have been proposed to attain MPP from a PV module. Among linear control techniques, the PID controller is normally used in literature and industrial plants due to its simplicity in implementation and low cost. These controllers have been utilized with numerous MPPT algorithms. In [23], the author optimized the PID controller parameters to fetch maximum power. Likewise, the PI controller gains for a standalone PV system were optimized using the ACO algorithm to get MPPT [24]. Gradient descent optimization (GDO) algorithm is used to optimize the PID controller gains for better performance [25]. Linear controllers show better performance but unable to effectively handle the system nonlinearities and operation over a wide range of operating conditions. These techniques are unreliable under varying conditions of the environment. It is essential to adapt the control strategy to handle nonlinearities and maintain performance under varying operating conditions.

In recent times, a lot of research work has been done in the nonlinear control area to achieve MPPT through different nonlinear control schemes. Several controllers are proposed to fetch maximum power like sliding mode control (SMC) [26] and backstepping [2]. The cell of a PV panel has a nonlinear nature. In [27], a nonlinear backstepping control technique is proposed to power up the towers of cellular networks. SMC method is widely used for nonlinear control systems. The control of an electronic converter is provided in this technique, which helps in tracking MPP under varying conditions of the environment. In the PV system, buck-boost converter transfers the power from panel to load. SMC mainly depends upon the switching frequency of the converter to obtain MPP. As the switching increases then speed of MPPT becomes faster. In [28], terminal sliding mode control (TSMC) is implemented to attain MPP for PV system with uncertainties. Similarly, backstepping based sliding mode (BSMC) controller is designed to obtain maximum power [29].

In the proposed work, RBF NN based BTSMC is proposed for MPPT of a PV system in varying environmental conditions. TSMC technique provides better results under varying atmospheric conditions. Backstepping technique [2] is highly efficient but not robust in varying conditions. For better robustness in varying conditions, Backstepping is synthesized with TSMC nonlinear control technique. When TSMC is compared with the other techniques, it offers fast convergence and better tracking precision in a finite time. BTSMC along RBF NN seems a better approach to extract maximum power due to less chattering effect, improve transient response, tracking with precision, and fast convergence.

**Fig 1:**
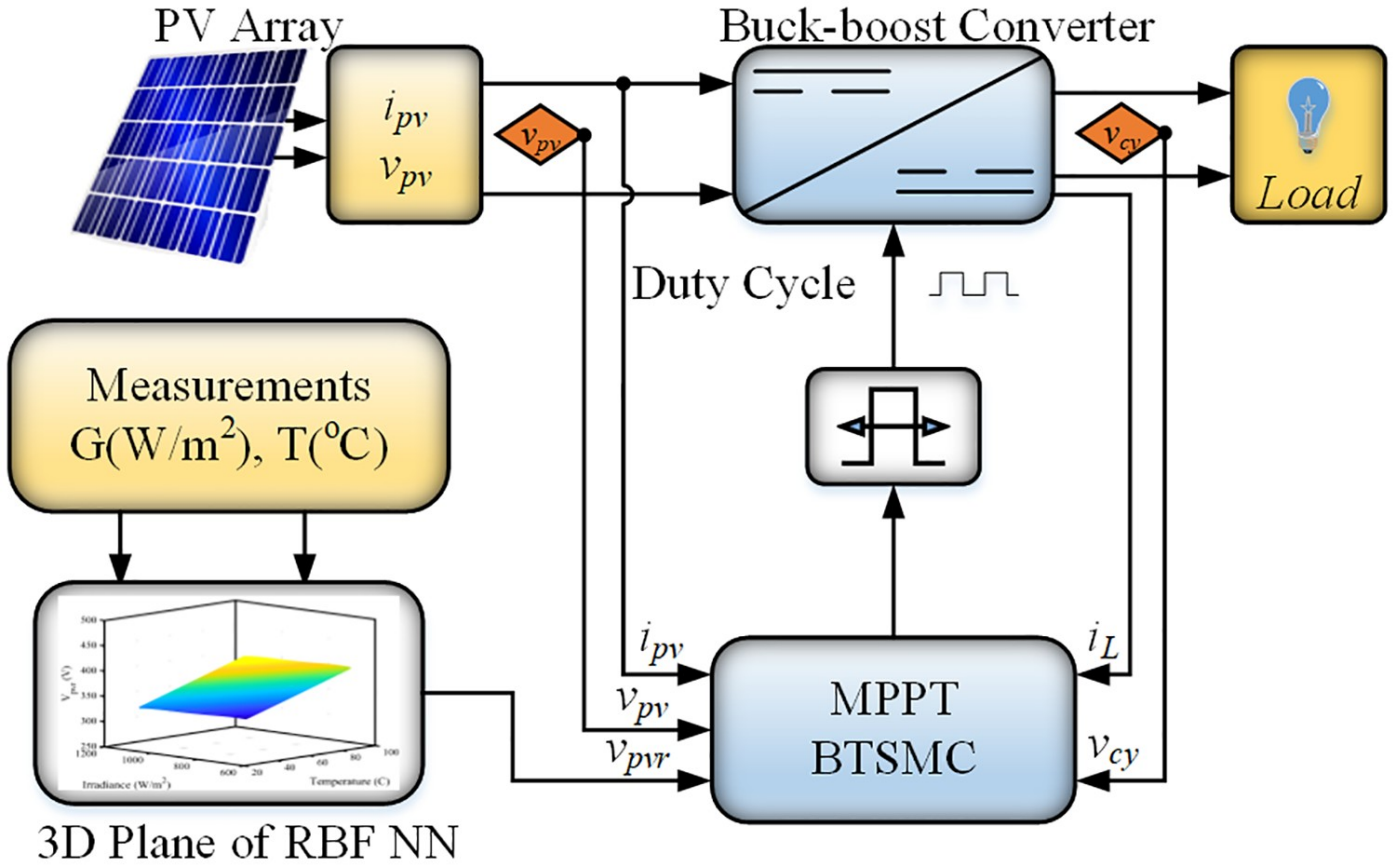
Block diagram of proposed work.

The working principle of the proposed algorithm is completely different when it is compared with the conventional algorithms. The proposed technique senses irradiance and temperature on the PV panel. The set of data is provided to the RBF NN, as presented in Fig 1 The RBF NN utilizes the relation between varying conditions of the environment i.e., Temperature and Irradiance and *V*_*mpp*_ to generate the reference voltage. The nonlinear controller can track the reference voltage *V*_*pvr*_. The controller is designed using a non-inverting converter mathematical model. The output signal of the controller controls the converter’s duty ratio through the pulse width modulation.

The paper structure is organized as: following an introduction in section 1, radial basis function neural network is described in section 2. The modeling of the converter is presented in section 3. The proposed control scheme with system stability analysis is explained in section 4. The performance of the controller is analyzed in section 5. In the end, section 6 concludes the findings of the research article.

## 2 Radial basis function neural network

**Fig 2:**
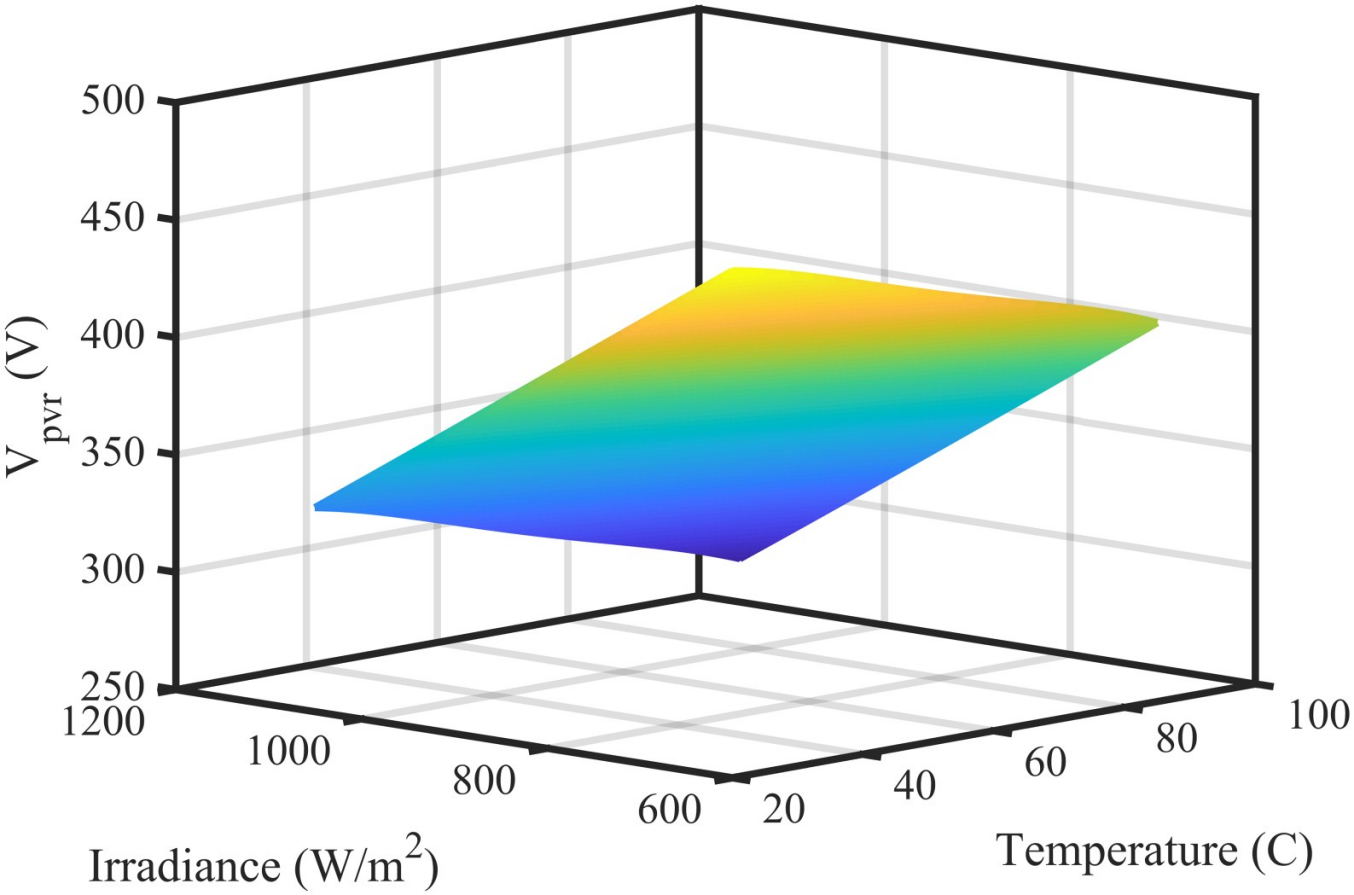
3-D plane of RBF NN.

**Fig 3:**
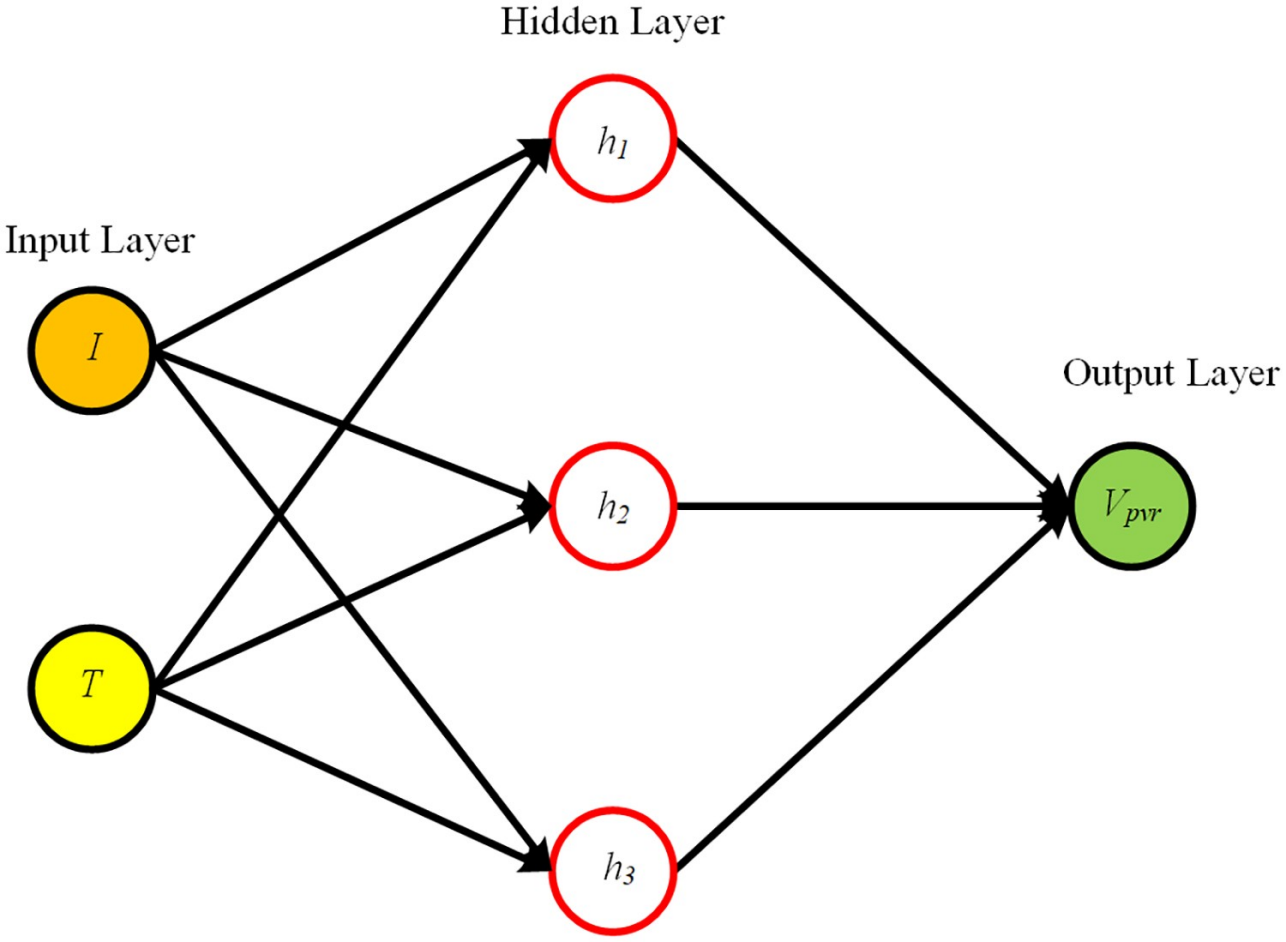
RBF NN inner view.

In this work, RBF NN generates reference voltage for the BTSMC. Recently, RBF NN has gained attention as compared to other feed-forward neural networks (FF NN) due to its simple structure and better generalization ability. The source nodes are present in the input layer which establishes a connection between the network and environment. In a hidden layer, a set of the activation functions is provided through hidden units. These units are hidden nodes. The activation function which produces the hidden layer output, is:

$$\begin{array}{c} \begin{array}{ccc} h_{j}\left(t\right)={\left(e\right)}^{-\frac{{∥x\left(t\right)-c_{j}\left(t\right)∥}^{2}}{2b_{j}^{2}}}, & & j=1,2,..,m \end{array} \end{array}$$ (1)

where *b*_*j*_ denotes positive scalar known as width and *m* denotes no. of hidden nodes. In proposed work, three hidden nodes are used in the hidden layer. The output layer which is the combination of linear weights, as follows:

$$\begin{array}{c} \begin{array}{ccccc} & y_{i}\left(t\right)=\sum_{j=1}^{m}w_{ji}h\left(t\right), & & & i=1,2,..,n \end{array} \end{array}$$ (2)

where *n* denotes no. of output, *w* denotes the weight of output layer and *y* denotes the output of the network. The result of the system is produced in an output layer which is the last layer of RBF NN. In this work, output of neural network is reference voltage *V*_*pvr*_. The data of reference voltage is obtained from characteristic curve of the PV module under various levels of irradiance (600 *W*/*m*^2^ − 1100 *W*/*m*^2^) and temperature (20°C–85°C). The data set having 29767 training points is used to train the neural network. The training data set has three entities: temperature, irradiance and voltage. Each entity has same number of training points. The 3-D plane of RBF NN obtained from this data is shown in Fig 2 while the inner view of the neural network is presented in Fig 3.

## 3 Non-inverting buck-boost DC-DC converter

**Fig 4:**
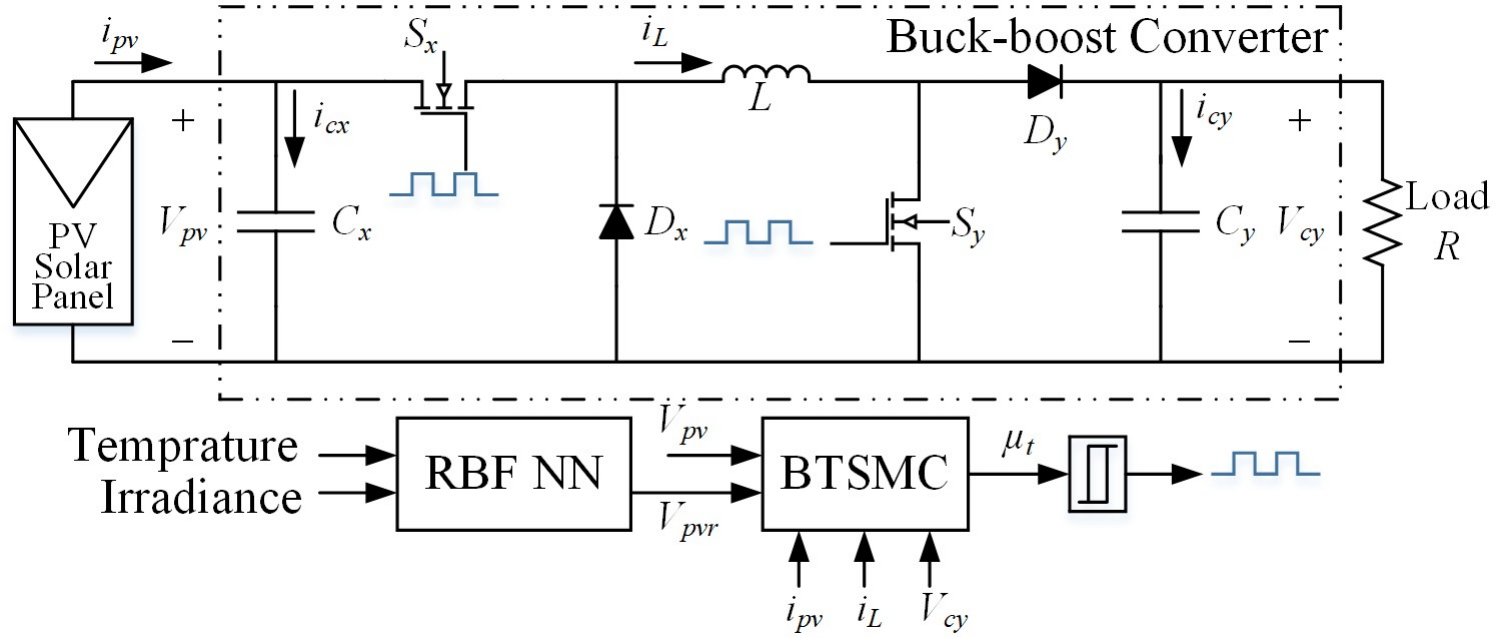
Non-inverting topology of buck-boost converter.

The non-inverting topology of buck-boost converter is utilized which has ability to step down or step up the voltage according to the requirement. This converter is used to follow the output voltage of PV array, *V*_*pv*_ to desired voltage, *V*_*mpp*_ through adjustment of duty cycle. The converter’s circuit diagram is presented in Fig 4.

The assumption has been made that converter is operated in continuous conduction mode. There are two modes of operation i.e. mode 1 and mode 2. In mode 1, both switches *S*_*x*_ and *S*_*y*_ are turned on and the diodes *D*_*x*_ and *D*_*y*_ are in reverse biased condition. In mode 2, the switches *S*_*x*_ and *S*_*y*_ are turned off while the diodes are forward biased. According to Fig 4, state-space equations for the first switching interval in vector-matrix form are:

$$\begin{array}{c} \left[\begin{array}{c} \frac{dv_{pv}}{dt}\\ \frac{di_{L}}{dt}\\ \frac{dv_{cy}}{dt} \end{array}]=[\begin{array}{ccc} 0 & -\frac{1}{C_{x}} & 0\\ \frac{1}{L} & 0 & 0\\ 0 & 0 & -\frac{1}{RC_{y}} \end{array}][\begin{array}{c} v_{pv}\\ v_{L}\\ v_{c_{y}} \end{array}]+[\begin{array}{c} \frac{I_{pv}}{C_{y}}\\ 0\\ 0 \end{array}\right] \end{array}$$ (3)

The state-space equations for the second switching interval in matrix form are as follows:

$$\begin{array}{c} \left[\begin{array}{c} \frac{dv_{pv}}{dt}\\ \frac{di_{L}}{dt}\\ \frac{dv_{c_{y}}}{dt} \end{array}]=[\begin{array}{ccc} 0 & 0 & 0\\ 0 & 0 & -\frac{1}{L}\\ 0 & \frac{1}{C_{y}} & -\frac{1}{RC_{y}} \end{array}][\begin{array}{c} v_{pv}\\ i_{L}\\ v_{c_{y}} \end{array}]+[\begin{array}{c} \frac{I_{pv}}{C_{x}}\\ 0\\ 0 \end{array}\right] \end{array}$$ (4)

Now the average model for non-inverting buck-boost converter in vector-matrix form is as follows:

$$\begin{array}{c} \left[\begin{array}{c} \frac{dv_{pv}}{dt}\\ \frac{di_{L}}{dt}\\ \frac{dv_{c_{y}}}{dt} \end{array}]=[\begin{array}{ccc} 0 & -\frac{u}{C_{x}} & 0\\ \frac{u}{L} & 0 & \left(\frac{u}{L}-\frac{1}{L}\right)\\ 0 & \left(\frac{1}{C_{y}}-\frac{u}{C_{y}}\right) & -\frac{1}{RC_{y}} \end{array}][\begin{array}{c} v_{pv}\\ i_{L}\\ v_{c_{y}} \end{array}]+[\begin{array}{c} \frac{I_{pv}}{C_{x}}\\ 0\\ 0 \end{array}\right] \end{array}$$ (5)

Assuming *x*_1_, *x*_2_, *x*_3_ and *μ* as the average values of *v*_*pv*_, *i*_*L*_, $v_{c_{y}}$ and *u*, respectively. Under these assumptions, then desired state-space model is presented as:

$$\begin{array}{c} \left[\begin{array}{c} {\dot{x}}_{1}\\ {\dot{x}}_{2}\\ {\dot{x}}_{3} \end{array}]=[\begin{array}{ccc} 0 & -\frac{\mu }{C_{x}} & 0\\ \frac{\mu }{L} & 0 & \left(\frac{\mu }{L}-\frac{1}{L}\right)\\ 0 & \left(\frac{1}{C_{y}}-\frac{\mu }{C_{y}}\right) & -\frac{1}{RC_{y}} \end{array}][\begin{array}{c} x_{1}\\ x_{2}\\ x_{3} \end{array}]+[\begin{array}{c} \frac{I_{pv}}{C_{x}}\\ 0\\ 0 \end{array}\right] \end{array}$$ (6)

## 4 Backstepping terminal sliding mode control design

A nonlinear BTSMC controller is designed to track *x*_1*r*_ = *V*_*pvr*_ for maximum power extraction. The backstepping is a nonlinear control methodology that is generally used as a part of control design. The controller output *μ*_*t*_ controls the duty ratio of the converter’s switches. Firstly, define the tracking error:

$$\begin{array}{c} e_{1}=x_{1}-x_{1r} \end{array}$$ (7)

Taking derivative of Eq (7),

$$\begin{array}{c} \dot{e_{1}}=\dot{x_{1}}-\dot{x_{1r}} \end{array}$$ (8)

Using dynamics of converter, we get

$$\begin{array}{c} \dot{e_{1}}=\frac{I_{pv}}{C_{x}}-\frac{x_{2}}{C_{x}}\mu -\dot{x_{1r}} \end{array}$$ (9)

Now introducing the stabilization function ‘*α*_1_’,

$$\begin{array}{c} \alpha_{1}=-\lambda_{1}e_{1}-\frac{e_{1}^{\frac{\Omega_{1}}{\Omega_{2}}}}{\lambda_{2}} \end{array}$$ (10)

where Ω_1_, Ω_2_ (1 < Ω_1_/Ω_2_ < 2) and λ_1_, λ_2_ are positive odd numbers. The tracking error has upgraded with the stabilization function.

$$\begin{array}{c} e_{2}={\dot{e}}_{1}-\alpha_{1} \end{array}$$ (11)

Applying first Lyapunov stability function,

$$\begin{array}{c} V_{1}=\frac{1}{2}e_{1}^{2} \end{array}$$ (12)

Eq (12) can be re-written as;

$$\begin{array}{c} {\dot{V}}_{1}=e_{1}e_{2}-\lambda_{1}e_{1}^{2}-\frac{e_{1}^{\left(\frac{\Omega_{1}+\Omega_{2}}{\Omega_{2}}\right)}}{\lambda_{2}} \end{array}$$ (13)

From Eq (11), then we get

$$\begin{array}{c} \begin{array}{ccc} \dot{e_{2}}= & \frac{I_{pv}}{c_{x}}-\frac{x_{2}}{c_{x}}\mu -\frac{\mu }{C_{x}}[-\frac{x_{3}}{L}+(\frac{x_{1}+x_{3}}{L})\mu ]-x_{1r}^{..}+\lambda_{1}{\dot{e}}_{1} & +\frac{\Omega_{1}}{\lambda_{2}\Omega_{2}}e_{1}^{\left(\frac{\Omega_{1}-\Omega_{2}}{\Omega_{2}}\right)}{\dot{e}}_{1} \end{array} \end{array}$$ (14)

Applying second Lyapunov stability function;

$$\begin{array}{c} V=V_{1}+\frac{1}{2}s^{T}s \end{array}$$ (15)

The sliding surface ‘*s*’ is followed as:

$$\begin{array}{c} s=e_{1}+e_{2} \end{array}$$ (16)

Taking the derivative of second lyapunov stability function;

$$\begin{array}{c} \dot{V}=e_{1}e_{2}-\lambda_{1}e_{1}^{2}-\frac{{\left(e_{1}\right)}^{\left(\frac{\Omega_{1}-\Omega_{2}}{\Omega_{2}}\right)}}{\lambda_{2}}+s^{T}[\dot{e_{1}}\left(1+\lambda_{1}\right)+\frac{\Omega_{1}}{\lambda_{2}\Omega_{2}}{\left(e_{1}\right)}^{\left(\frac{\Omega_{1}-\Omega_{2}}{\Omega_{2}}\right)}\dot{e_{1}}+\frac{\dot{I_{pv}}}{C_{x}}-\\ {\dot{\mu }}_{t}\frac{x_{2}}{C_{x}}-\frac{\mu }{C_{x}}(\frac{-x_{3}}{L}+(\frac{x_{1}+x_{3}}{L})\mu )-\ddot{x_{1r}})] \end{array}$$ (17)

Total control law is characterized as:

$$\begin{array}{c} \dot{\mu_{t}}=\dot{\mu_{eq.}}+\dot{\mu_{dis.}} \end{array}$$ (18)

Substituting Eq (18) in Eq (17);

$$\begin{array}{c} \dot{V}=e_{1}e_{2}-\lambda_{1}e_{1}^{2}-\frac{{\left(e_{1}\right)}^{\left(\frac{\Omega_{1}-\Omega_{2}}{\Omega_{2}}\right)}}{\lambda_{2}}+s^{T}[\dot{e_{1}}\left(1+\lambda_{1}\right)+\frac{\Omega_{1}}{\lambda_{2}\Omega_{2}}{\left(e_{1}\right)}^{\left(\frac{\Omega_{1}-\Omega_{2}}{\Omega_{2}}\right)}\dot{e_{1}}+\frac{\dot{I_{pv}}}{C_{x}}-{\dot{\mu }}_{eq.}\frac{x_{2}}{C_{x}}\\ -{\dot{\mu }}_{dis.}\frac{x_{2}}{C_{x}}-\frac{\mu }{C_{x}}(\frac{-x_{3}}{L}+(\frac{x_{1}+x_{3}}{L})\mu )-\ddot{x_{1r}})] \end{array}$$ (19)

$\dot{s}=0$ gives the equivalent control law, $\dot{\mu_{eq.}}$. This control law is very essential for desired tracking without any consideration of uncertainties and disturbances i.e. $\dot{\mu_{dis.}}$.

$$\begin{array}{c} {\dot{\mu }}_{eq.}=(\frac{C_{x}}{x_{2}})[\dot{e_{1}}\left(1+\lambda_{1}\right)+\frac{\Omega_{1}}{\lambda_{2}\Omega_{2}}{\left(e_{1}\right)}^{\left(\frac{\Omega_{1}-\Omega_{2}}{\Omega_{2}}\right)}\dot{e_{1}}+\frac{\dot{I_{pv}}}{C_{x}}-\frac{\mu }{C_{x}}(\frac{-x_{3}}{L}+(\frac{x_{1}+x_{3}}{L})\mu )-\ddot{x_{1r}}] \end{array}$$ (20)

Using Eqs (19) and (20), the expression for $\dot{V}$ is,

$$\begin{array}{c} \dot{V}=e_{1}e_{2}-\lambda_{1}e_{1}^{2}-\frac{e_{1}^{\left(\frac{\Omega_{1}+\Omega_{2}}{\Omega_{2}}\right)}}{\lambda_{2}}+s^{T}(\frac{-{\dot{\mu }}_{dis}x_{2}}{c_{x}}) \end{array}$$ (21)

To satisfy the condition of Lyapunov stability, the corrective control law ‘${\dot{\mu }}_{dis}$’ is described as:

$$\begin{array}{c} {\dot{\mu }}_{dis.}=\frac{C_{x}}{x_{2}}[\frac{1}{s^{T}}(e_{1}e_{2}-\frac{e_{1}^{\left(\frac{\Omega_{1}+\Omega_{2}}{\Omega_{2}}\right)}}{\lambda_{2}}+k\;sign\left(s\right))] \end{array}$$ (22)

Subtitution of Eq (22) in Eq (21);

$$\begin{array}{c} \dot{V}=-\lambda_{1}e_{1}^{2}-ks^{T}sign\left(s\right) \end{array}$$ (23)

where ‘*k*’ is sliding gain

$$\begin{array}{c} \dot{V}\leq \lambda_{1}|e_{1}^{2}|-k\left|s\right| \end{array}$$ (24)

where |*s*| = *s*^*T*^ *sign*(*s*), ‘*sign*’ function is replaced by tangent hyperbolic function, ‘tanh’. Signum function causes chattering phenomenon, therefore term ‘tanh’ is introduced to diminish this impact.

$$\begin{array}{c} \dot{V}\leq \lambda_{1}|e_{1}^{2}|-ks^{T}\tanh\left(s\right) \end{array}$$ (25)

The term ‘*s*^*T*^tanh(*s*)’ in Eq (25) is constantly positive so that condition has to be negative (i.e. *s*^*T*^tanh(*s*)>0 if either *s* > 0 or *s* < 0). Therefore achieving control signal is characterized as:

$$\begin{array}{c} {\dot{\mu }}_{dis}=\frac{C_{x}}{x_{2}}[\frac{1}{s^{T}}(e_{1}e_{2}-\frac{e_{1}^{\left(\frac{\Omega_{1}+\Omega_{2}}{\Omega_{2}}\right)}}{\lambda_{2}}+\tanh\left(s\right))] \end{array}$$ (26)

For total BTSMC control law, substituting Eqs (20) and (26) in Eq (18),

$$\begin{array}{c} \dot{\mu_{t}}=(\frac{C_{x}}{x_{2}})[\dot{e_{1}}\left(1+\lambda_{1}\right)+\frac{\Omega_{1}}{\lambda_{2}\Omega_{2}}{\left(e_{1}\right)}^{\left(\frac{\Omega_{1}-\Omega_{2}}{\Omega_{2}}\right)}\dot{e_{1}}+\frac{\dot{I_{pv}}}{C_{x}}-\frac{\mu }{C_{x}}(\frac{-x_{3}}{L}+(\frac{x_{1}+x_{3}}{L})\mu )-\ddot{x_{1r}}]\\ +\frac{C_{x}}{x_{2}}[\frac{1}{s^{T}}(e_{1}e_{2}-\frac{e_{1}^{\left(\frac{\Omega_{1}+\Omega_{2}}{\Omega_{2}}\right)}}{\lambda_{2}}+k\;\tanh\left(s\right))] \end{array}$$ (27)

**Fig 5:**
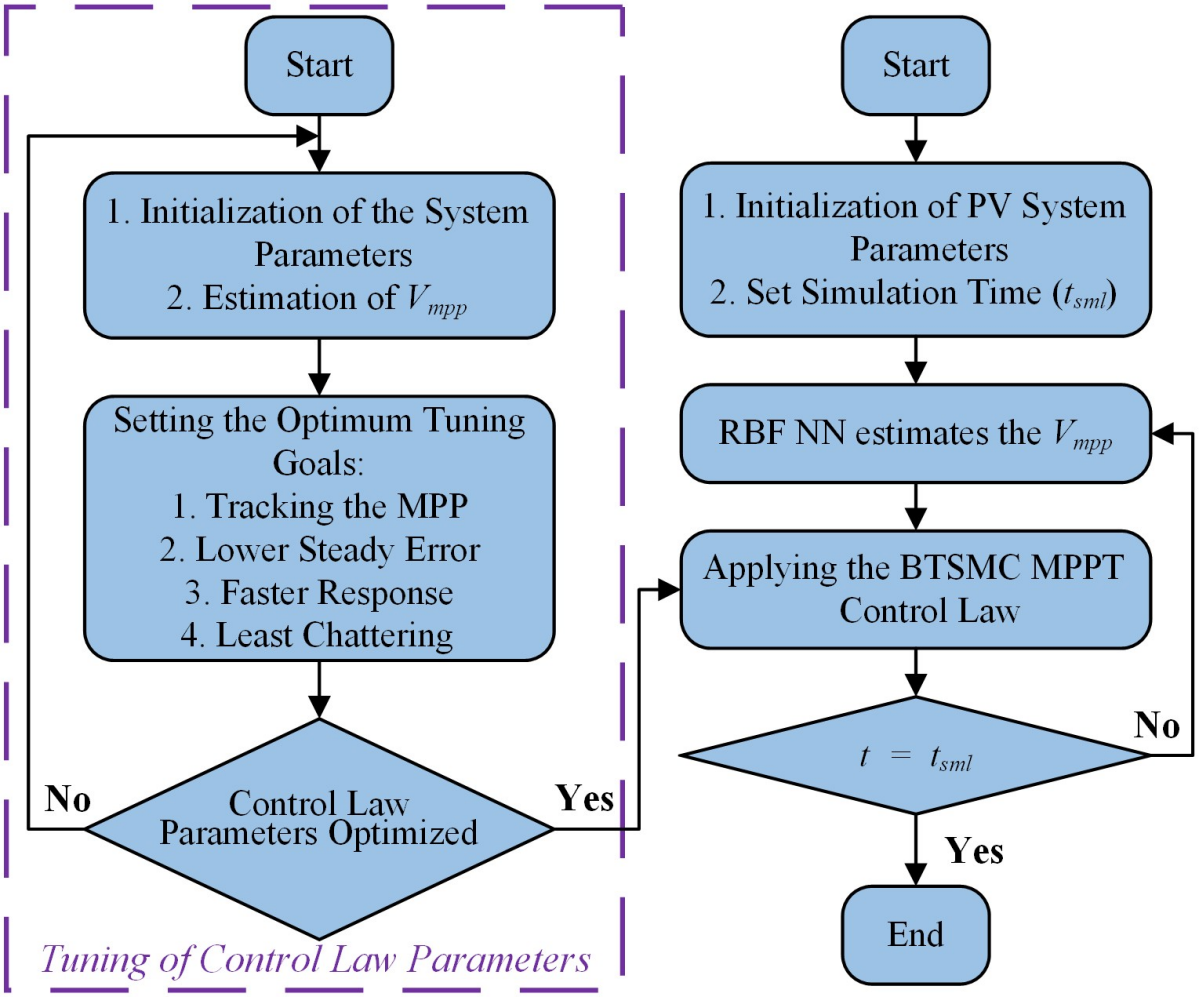
Flow chart of proposed technique.

**Fig 6:**
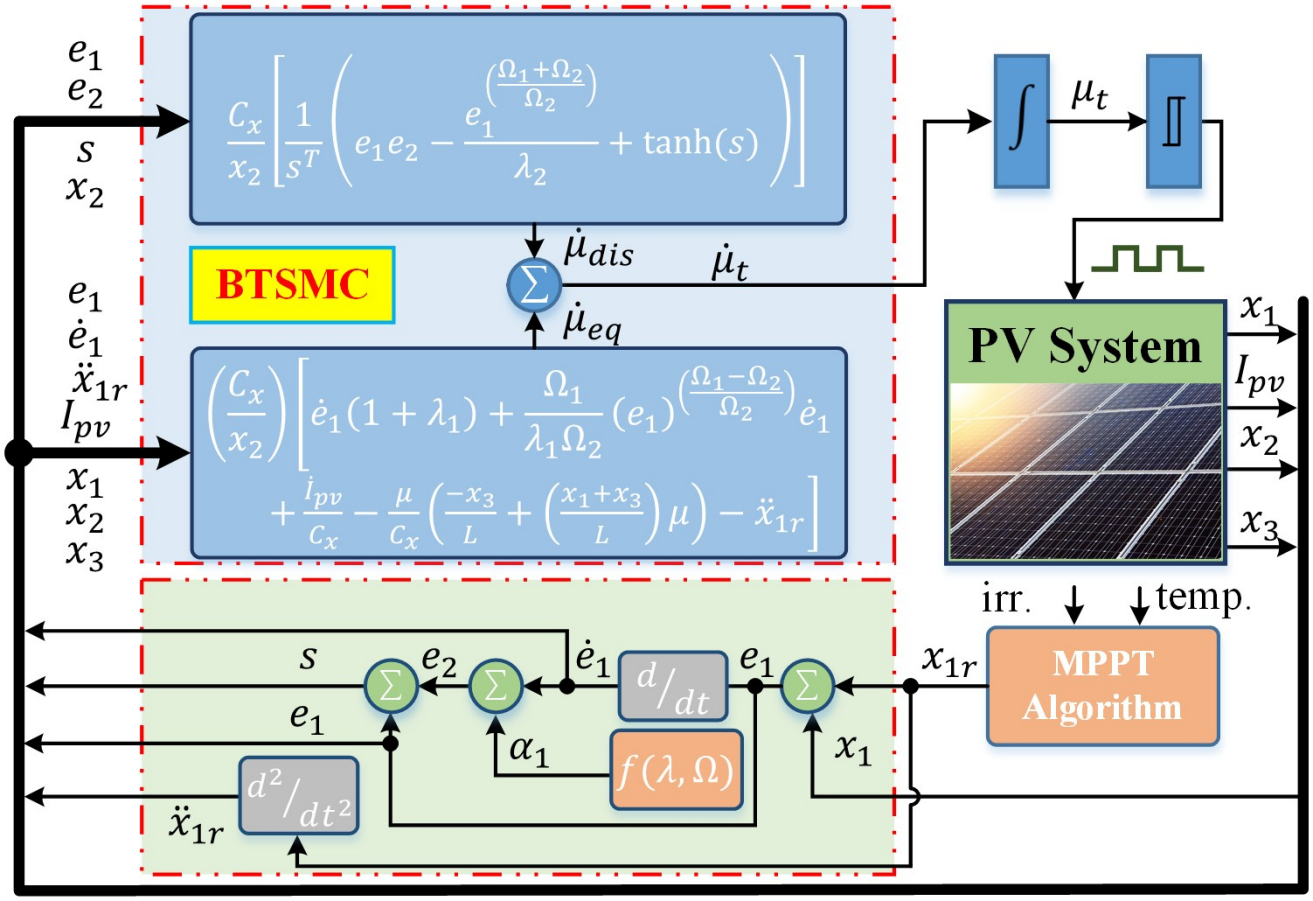
Closed-loop control system.

The flow chart and closed-loop system for the proposed nonlinear control paradigm is presented in Figs 5 and 6, respectively.

## 5 Simulation results and discussion

The performance of the proposed BTSMC scheme is validated through the simulations performed in MATLAB/Simulink. The parameters of the PV system are presented in Table 1, and the BTSMC and PID control parameter are mentioned in Table 2. Firstly, the BTSMC performance is analyzed under varying temperature and irradiance with varying load. Secondly, the performance of the proposed controller is benchmarked against backstepping control, conventional PID control and P&O algorithm. To further investigate the performance, BTSMC technique is compared with above mentioned techniques with fault and uncertainty in the system.

**Table 1:** Parameters of the PV system.

Name	Quantity	Value
PV Array	Series cells/PV module	72
	Parallel cells/PV module	1
	No. of modules/PV string	4
	No. of strings/PV array	4
	No. of modules/PV array	16
	Single module output power	$\begin{array}{c} 1,555\text{W}\\ 24,880\text{W}\\ 102.60\text{V}\\ 15.16\text{A}\\ 165.80\text{V}\\ 17.56\text{A} \end{array}\}@\text{STC}$
	PV array output power	
	Module voltage at MPP	
	Module current at MPP	
	Module open-circuit voltage	
	Module short-circuit current	
DC-DC Converter	Input capacitor, *C*_*x*_	1 *mF*
	Output capacitor, *C*_*y*_	48 *μF*
	Inductor, *L*	0.5 *mH*
	Switching frequency, *f*_*s*_	5 *kHz*
	Load resistances, *R*_*L*_	30, 40, 50 Ω

**Table 2:** Parameters of MPPT controllers.

Name	Gains	Value
Backstepping	Constant, *k*_1_	100
	Constant, *k*_2_	9000
PID	Constant, *k*_*P*_	0.002054
	Constant, *k*_*I*_	0.2737
	Constant, *N*	252.10
BTSMC	Constant, *k*	5
	Constant, Ω_1_	91
	Constant, Ω_2_	47.77
	Constant, λ_1_	9345
	Constant, λ_2_	1500

### 5.1 Performance comparison of proposed controller with backstepping, P&O and PID under varying load and environmental conditions

**Fig 7:**
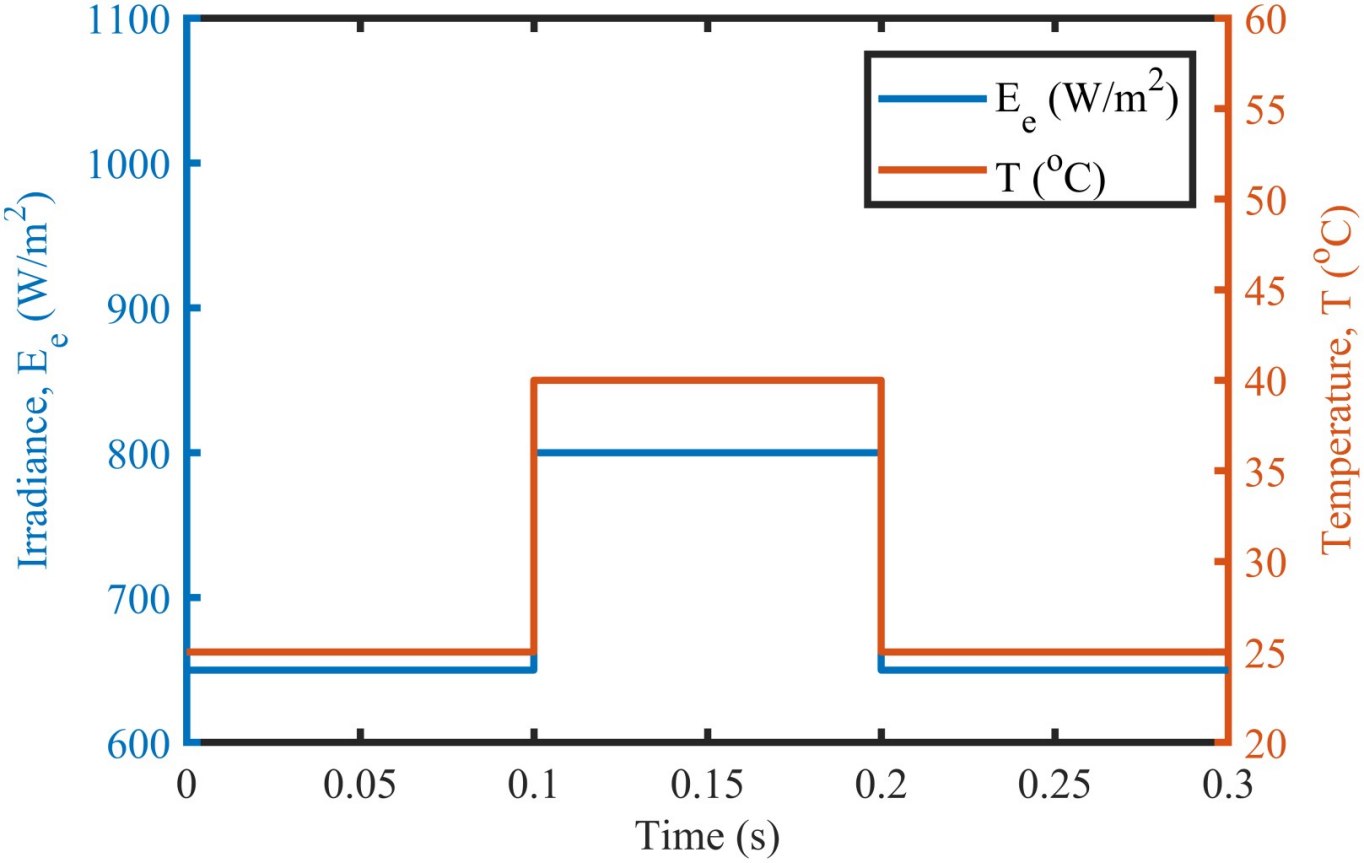
Varying meteorological conditions.

**Fig 8:**
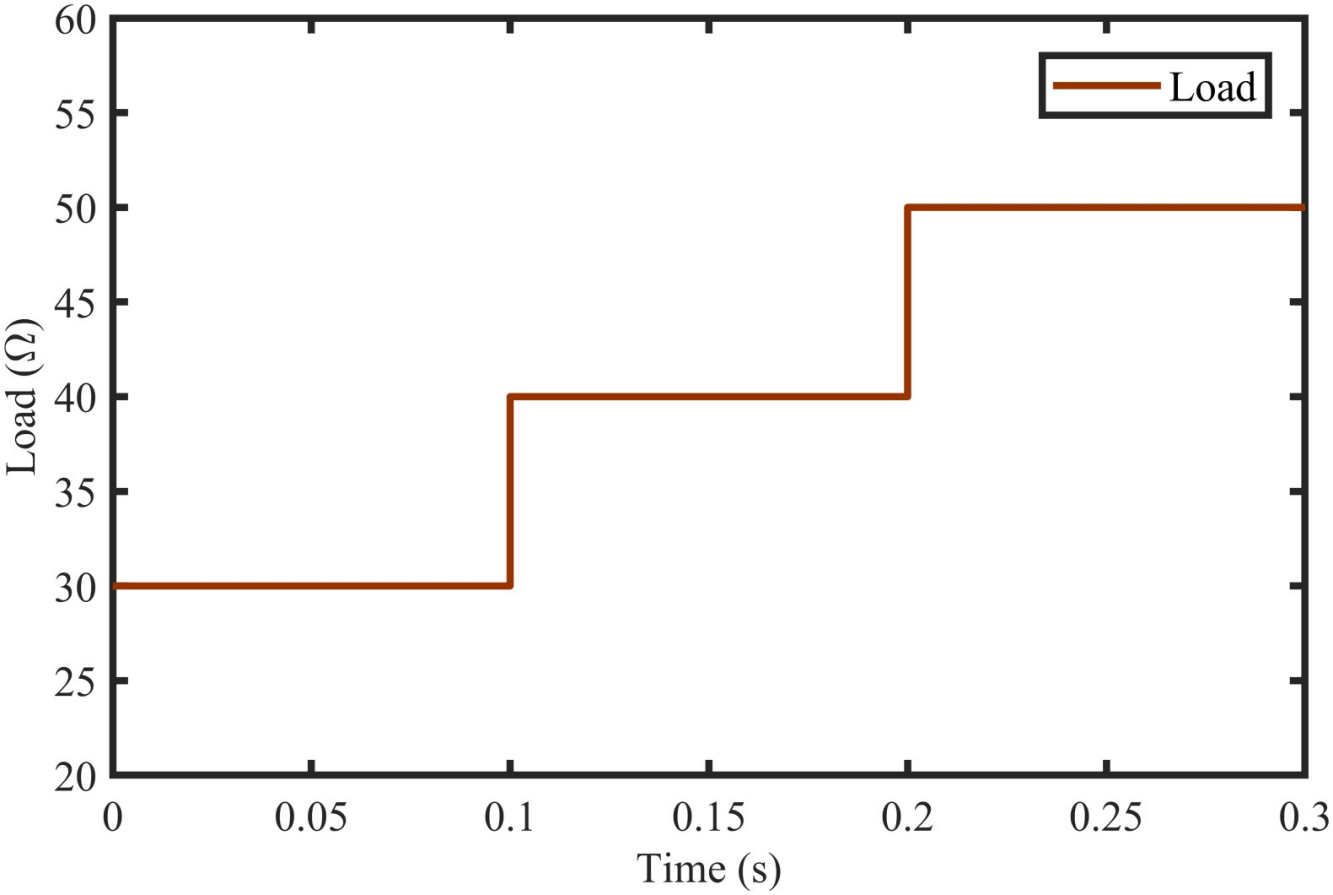
Scenario of varying load.

**Fig 9:**
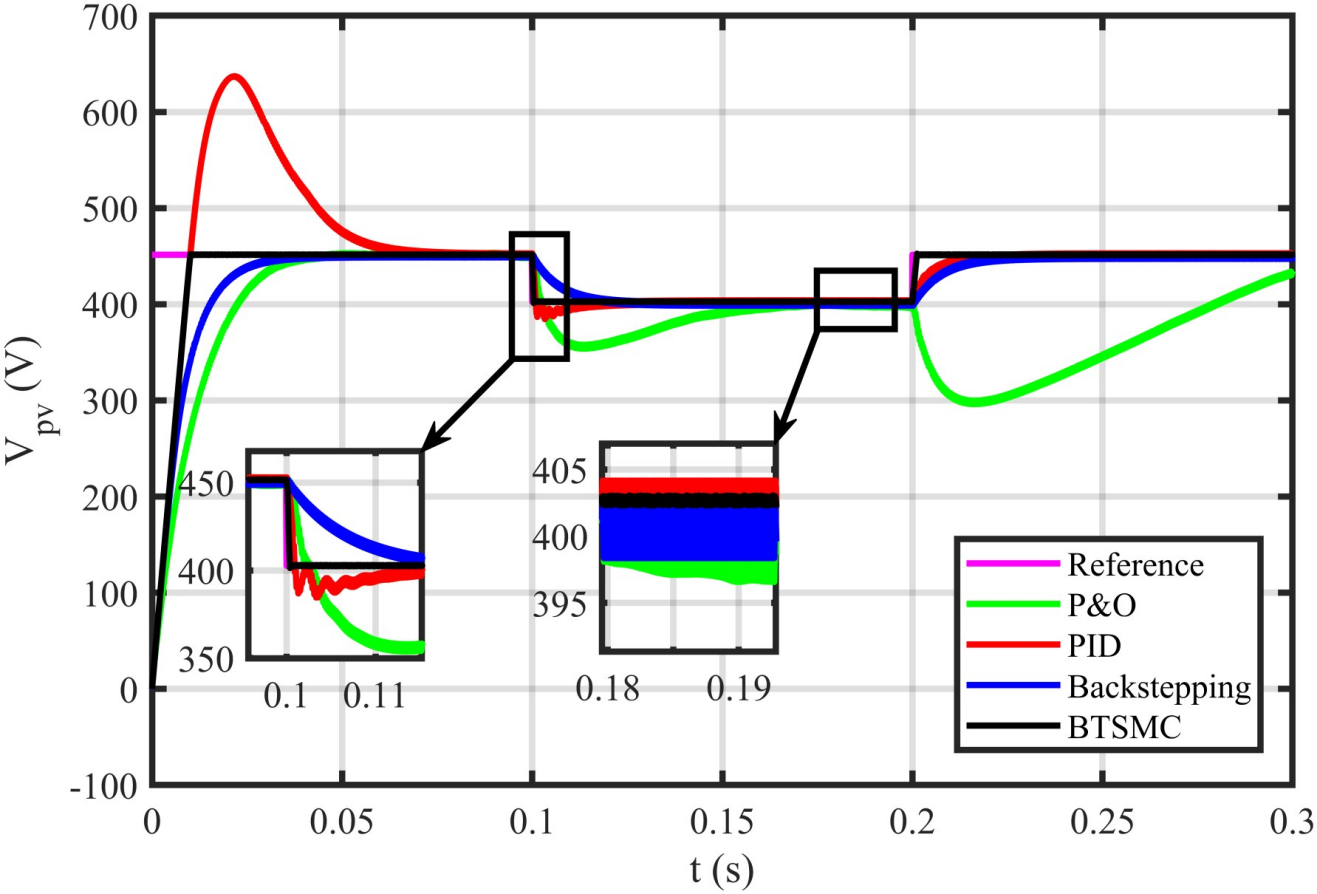
Output voltage under varying conditions.

**Fig 10:**
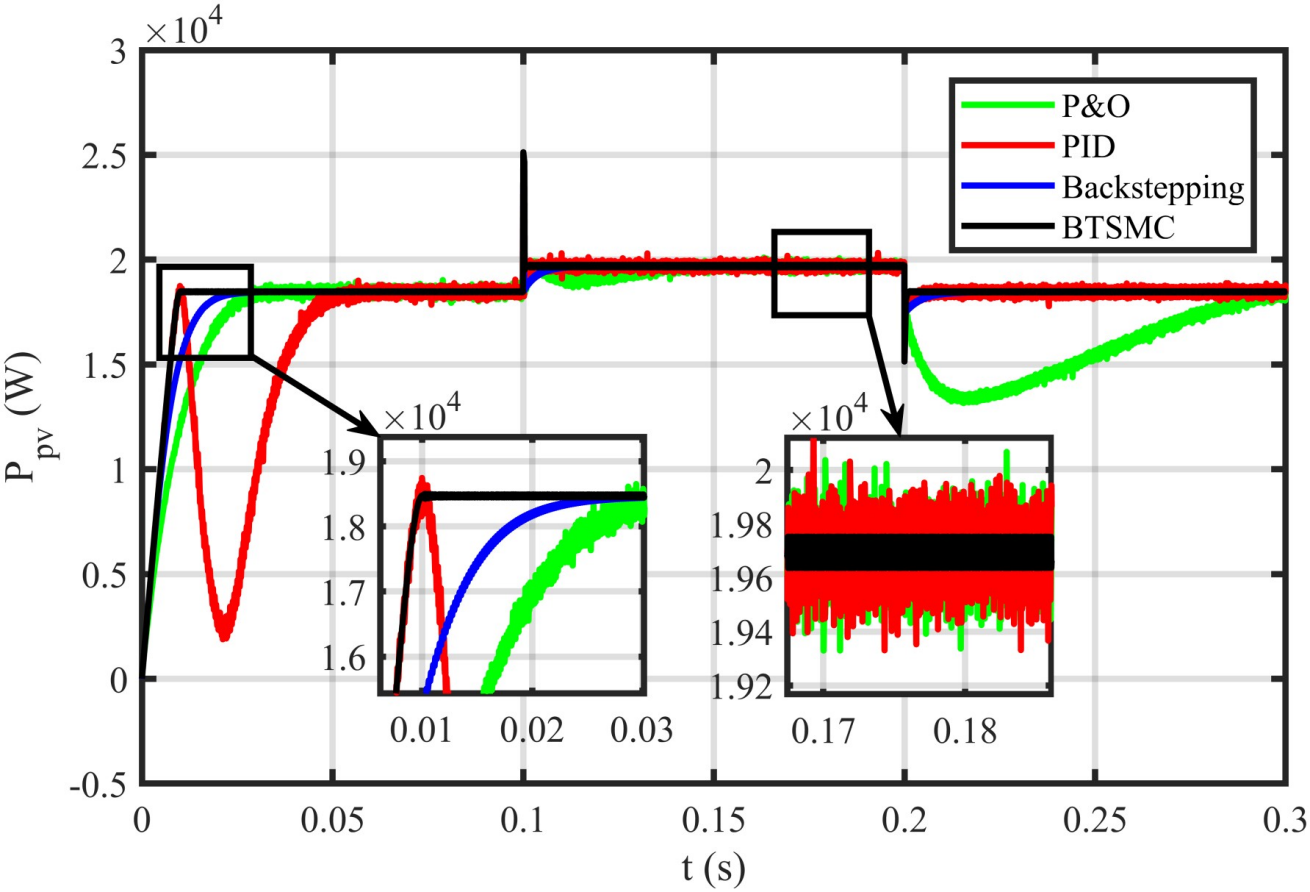
Output power under varying conditions.

The BTSMC performance is verified in the case of varying levels of irradiance and temperature, as shown in Fig 7 with varying residential load. Initially, level of irradiance and temperature are at 650 W/m^2^ and 25°C, respectively from 0.0 to 0.1 seconds and load at 30 Ω. Furthermore, conditions are changed to 800 W/m^2^ and 40°C from 0.1 to 0.2 seconds and load at 40 Ω. For the time interval of 0.2 to 0.3 seconds, the level of irradiance and temperature are again shifted to 650 W/m^2^ and 25°C, respectively and load at 50 Ω. The scenario of varying residential load is shown in Fig 8. The reference of voltage for varying environmental conditions is generated by RBF NN. The proposed controller performance with varying residential load is compared with P&O, PID, and backstepping controller under varying levels of irradiance and temperature. It is observed that BTSMC controller reach steady-state condition more rapidly at all levels as compared to the existing techniques as shown in Fig 9. The output power of the PV array is presented in Fig 10 with other MPP curves of contenders. It can also be observed that the MPP is attained successfully with negligible oscillations, as compared to other MPPT techniques.

**Fig 11:**
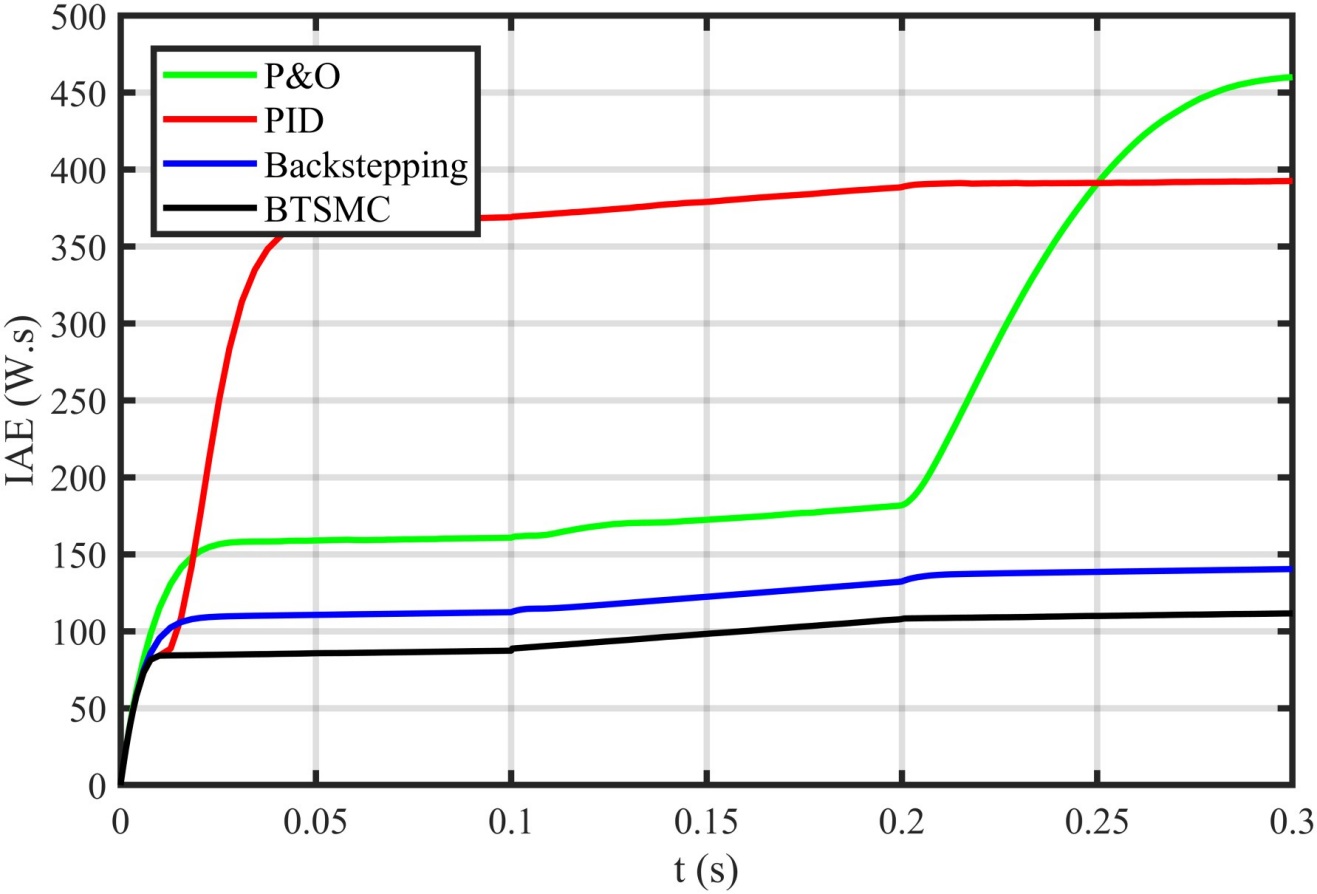
Performance index IAE under varying conditions.

**Fig 12:**
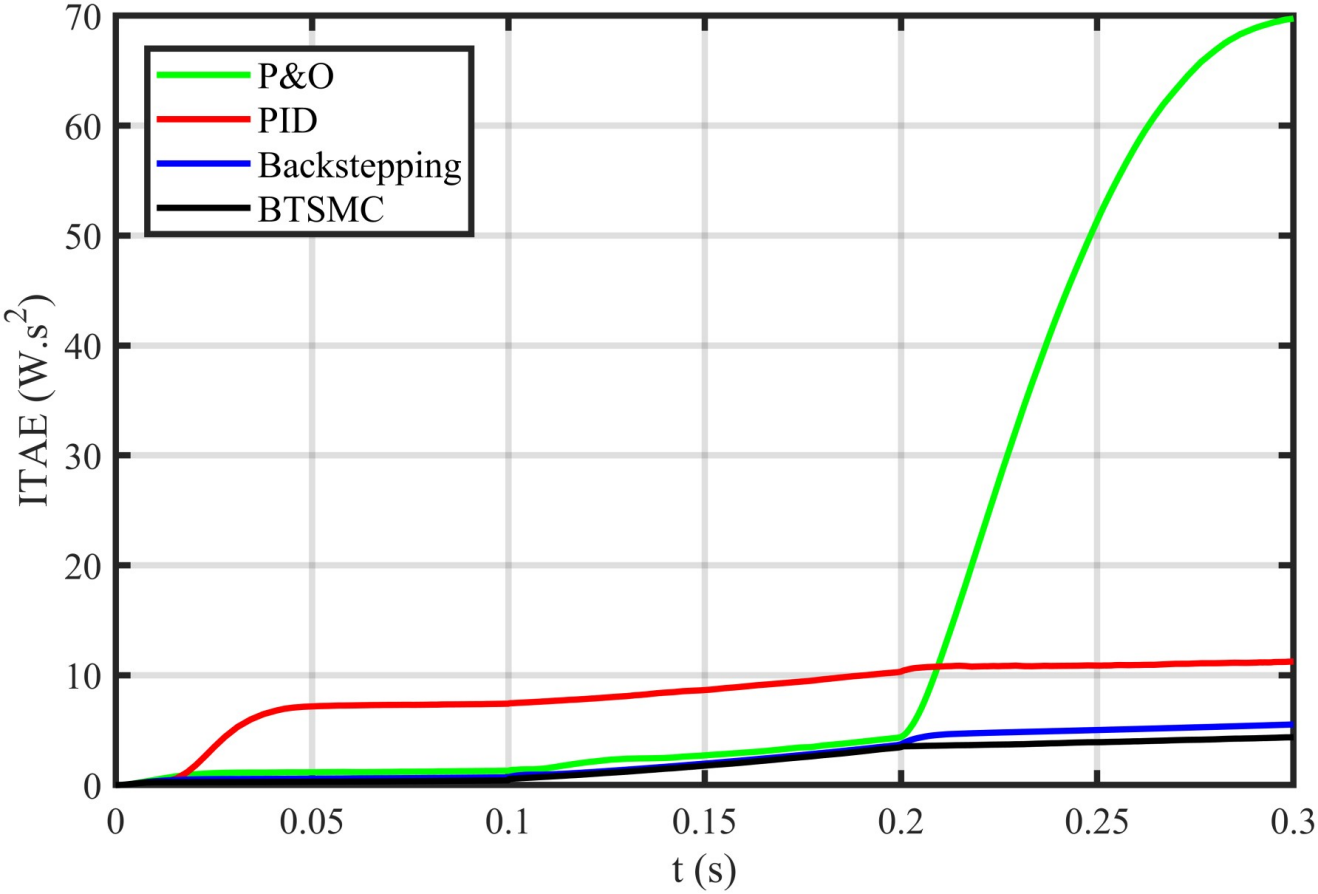
Performance index ITAE under varying conditions.

**Fig 13:**
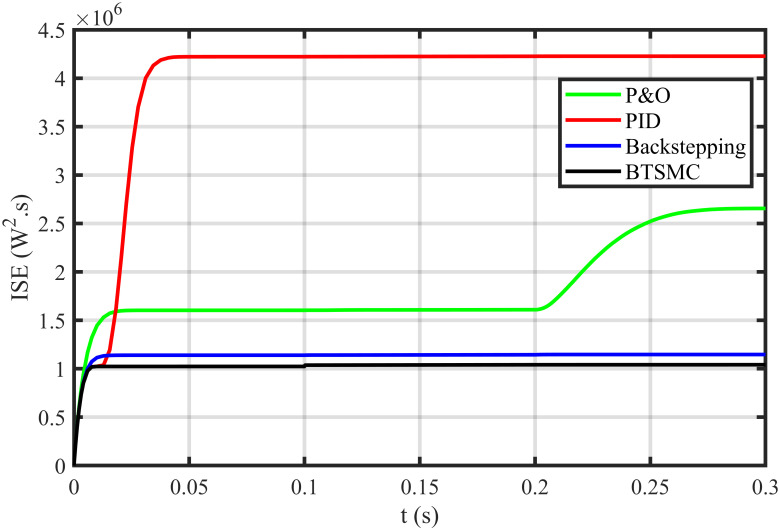
Performance index ISE under varying conditions.

**Fig 14:**
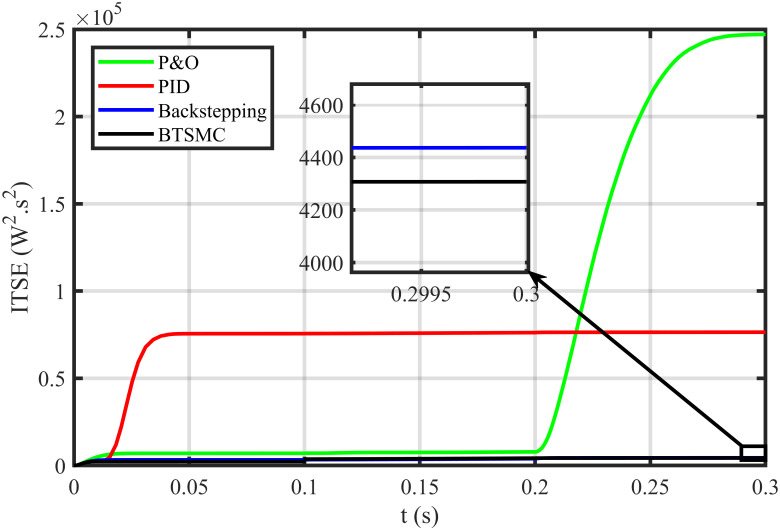
Performance index ITSE under varying conditions.

**Fig 15:**
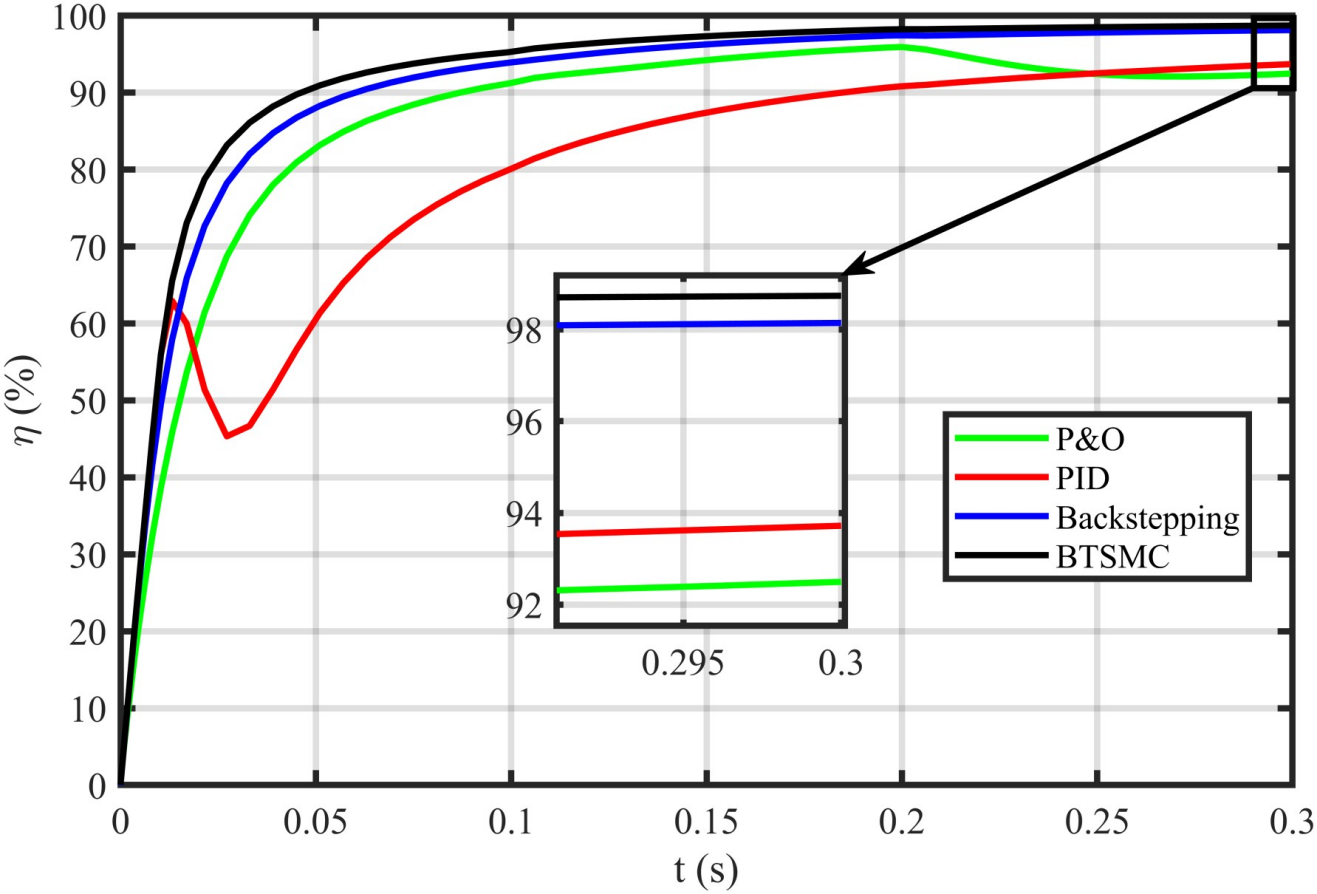
Efficiency (*η*) under varying conditions.

The error based four performance indices are also analyzed [30]. The indexes include integral of absolute error $(\text{IAE})=\int_{0}^{T}|e_{p}|dt$, integral of time-weighted absolute error $(\text{ITAE})=\int_{0}^{T}t|e_{p}|dt$, integral of squared error $(\text{ISE})=\int_{0}^{T}{\left(e_{p}\right)}^{2}dt$, and integral of time-weighted squared error $(\text{ITSE})=\int_{0}^{T}t{\left(e_{p}\right)}^{2}dt$. IAE, ITAE, ISE, and ITSE with the proposed controller and its other competitors are shown in Figs 11–14. In all indices, the BTSMC scheme shows the least index value that verifies the best performance as compared to the benchmarked control schemes. Fig 15 shows the conversion efficiency of the converter. The maximum power is extracted for the load by the developed BTSMC technique with the efficiency, *η* = 98.74%, which is maximum as compared to the existing MPPT techniques efficiencies. In this regard, BTSMC is robust under varying loads and meteorological conditions.

### 5.2 Comparison of proposed controller with P&O, PID and backstepping under varying climatic and faulty conditions

**Fig 16:**
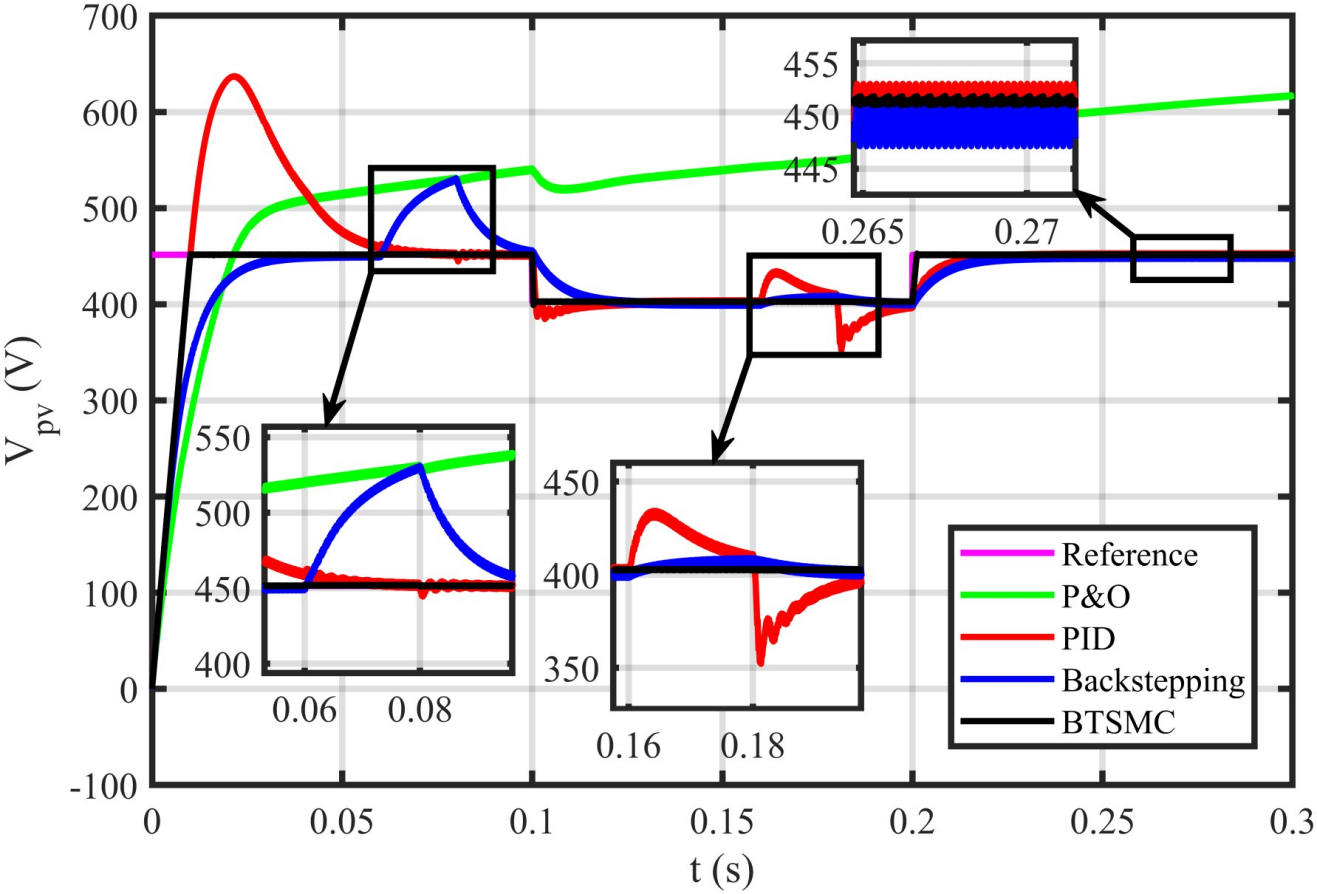
Output voltage under varying climatic and faulty conditions.

**Fig 17:**
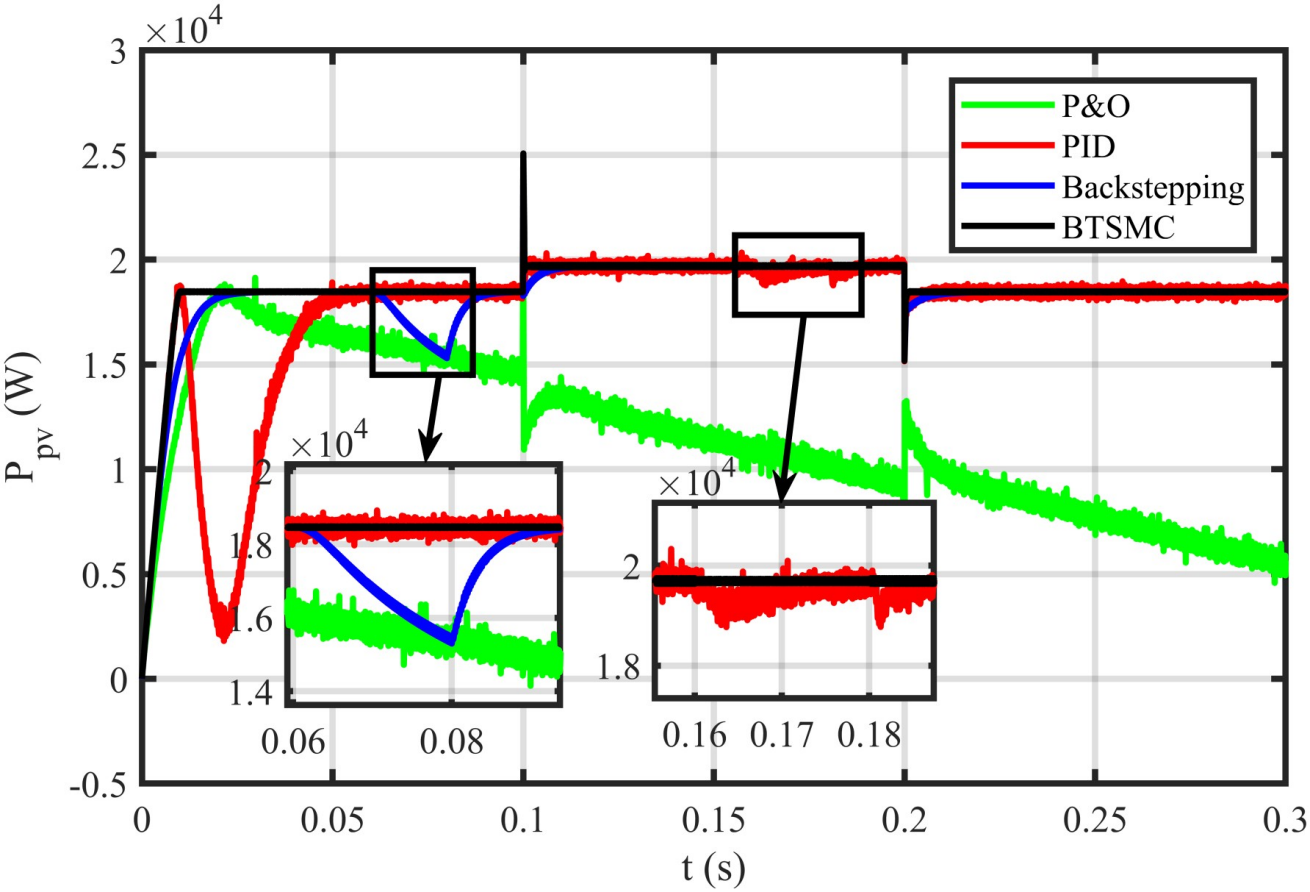
Output power under varying climatic and faulty conditions.

**Fig 18:**
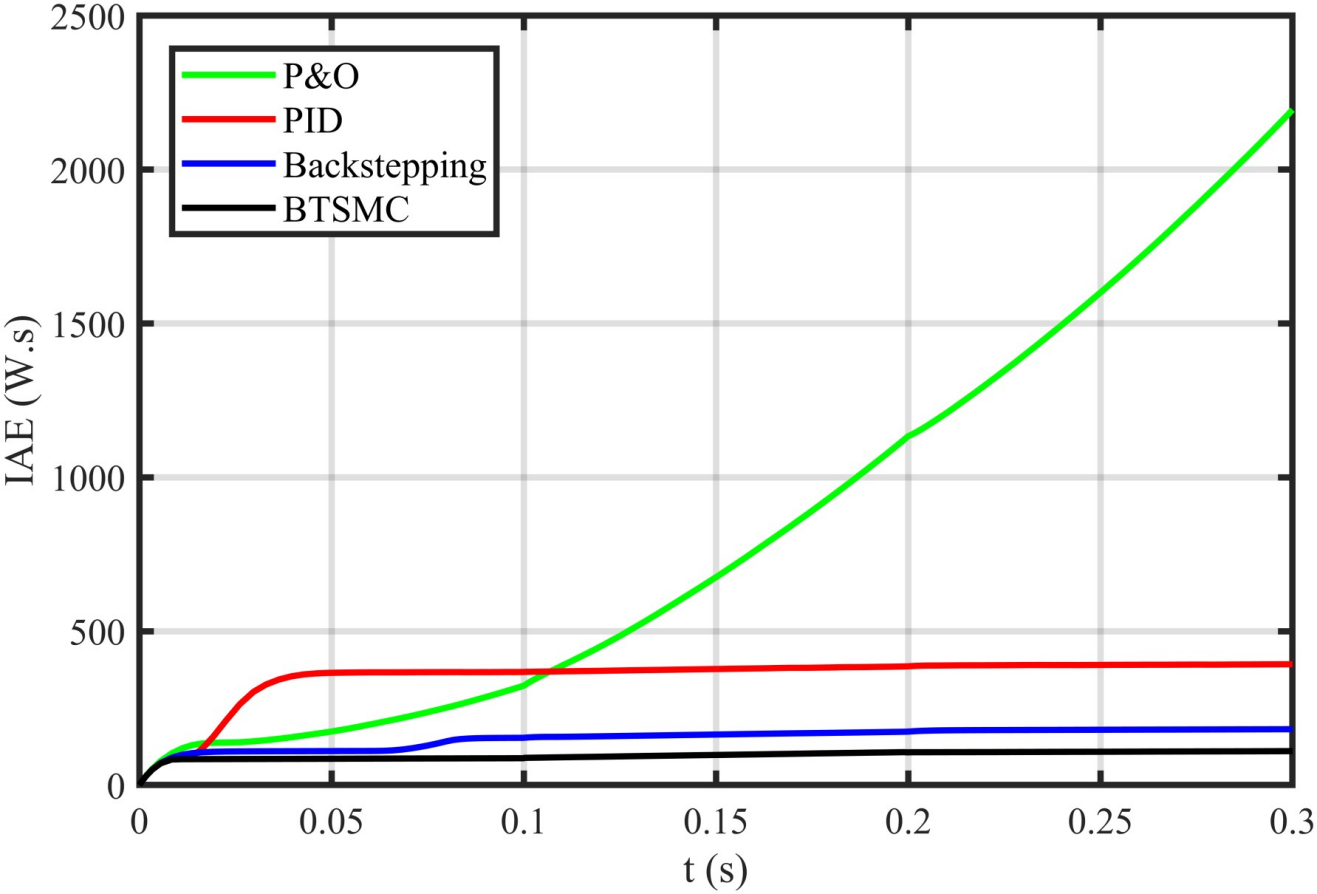
Performance index IAE under varying climatic and faulty conditions.

**Fig 19:**
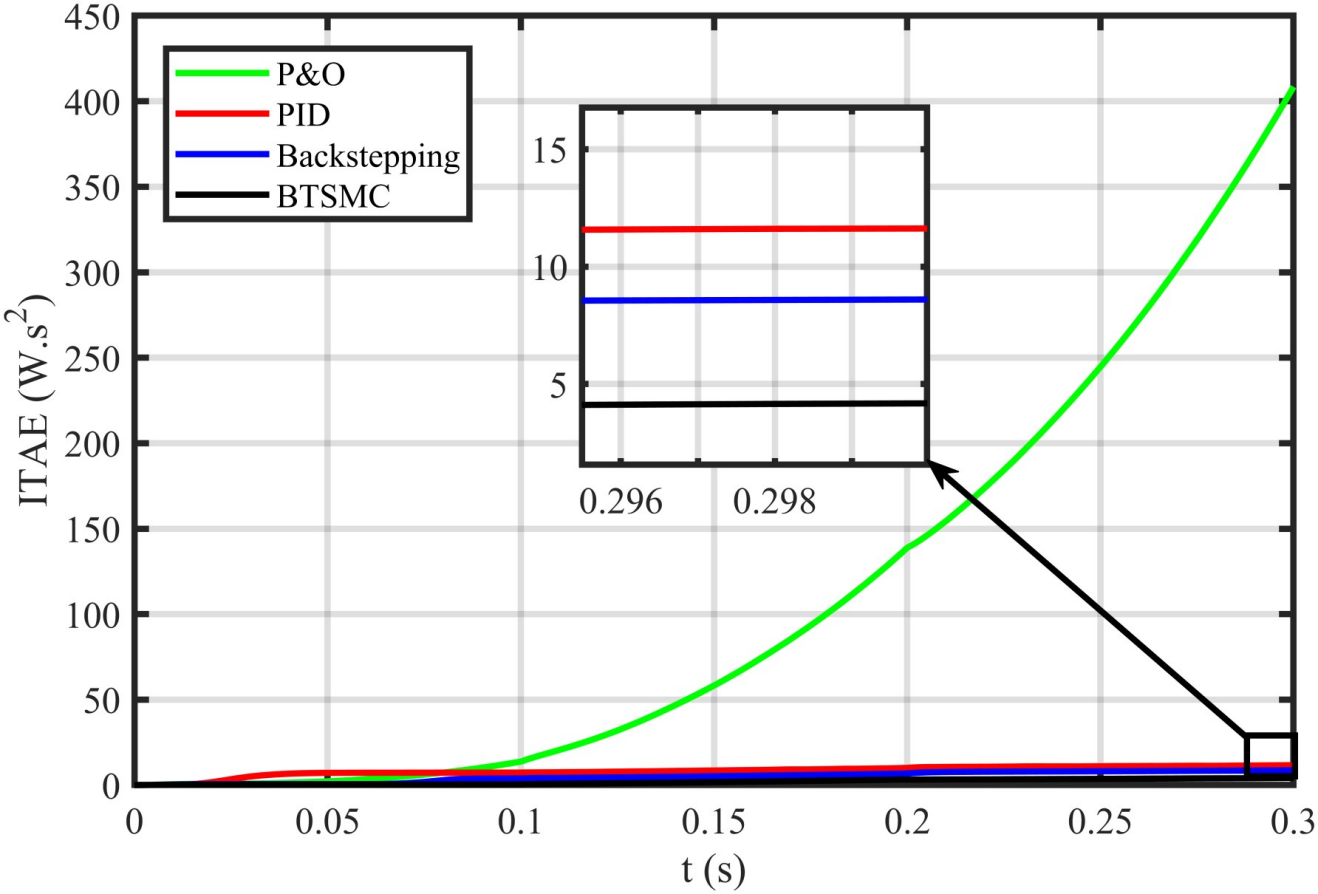
Performance index ITAE under varying conditions and faulty conditions.

**Fig 20:**
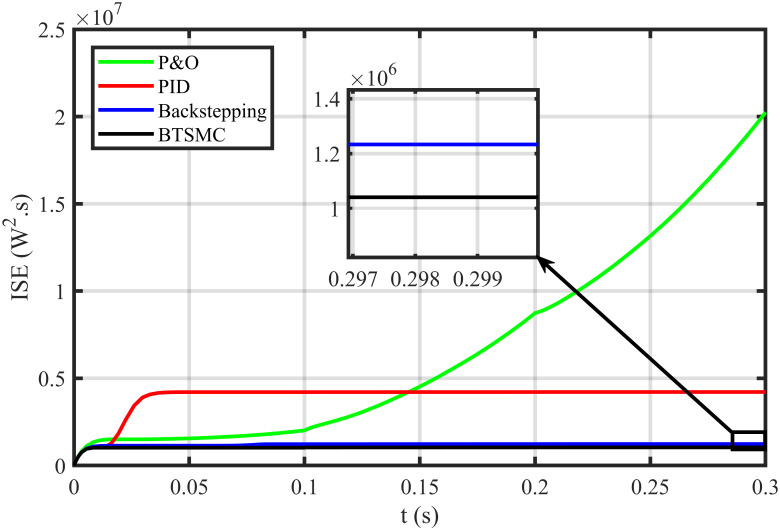
Performance index ISE under varying conditions and faulty conditions.

**Fig 21:**
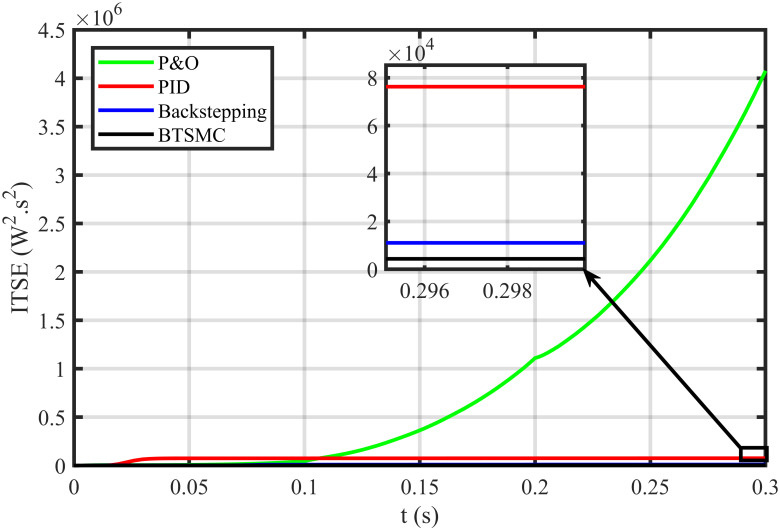
Performance index ITSE under varying conditions and faulty conditions.

**Fig 22:**
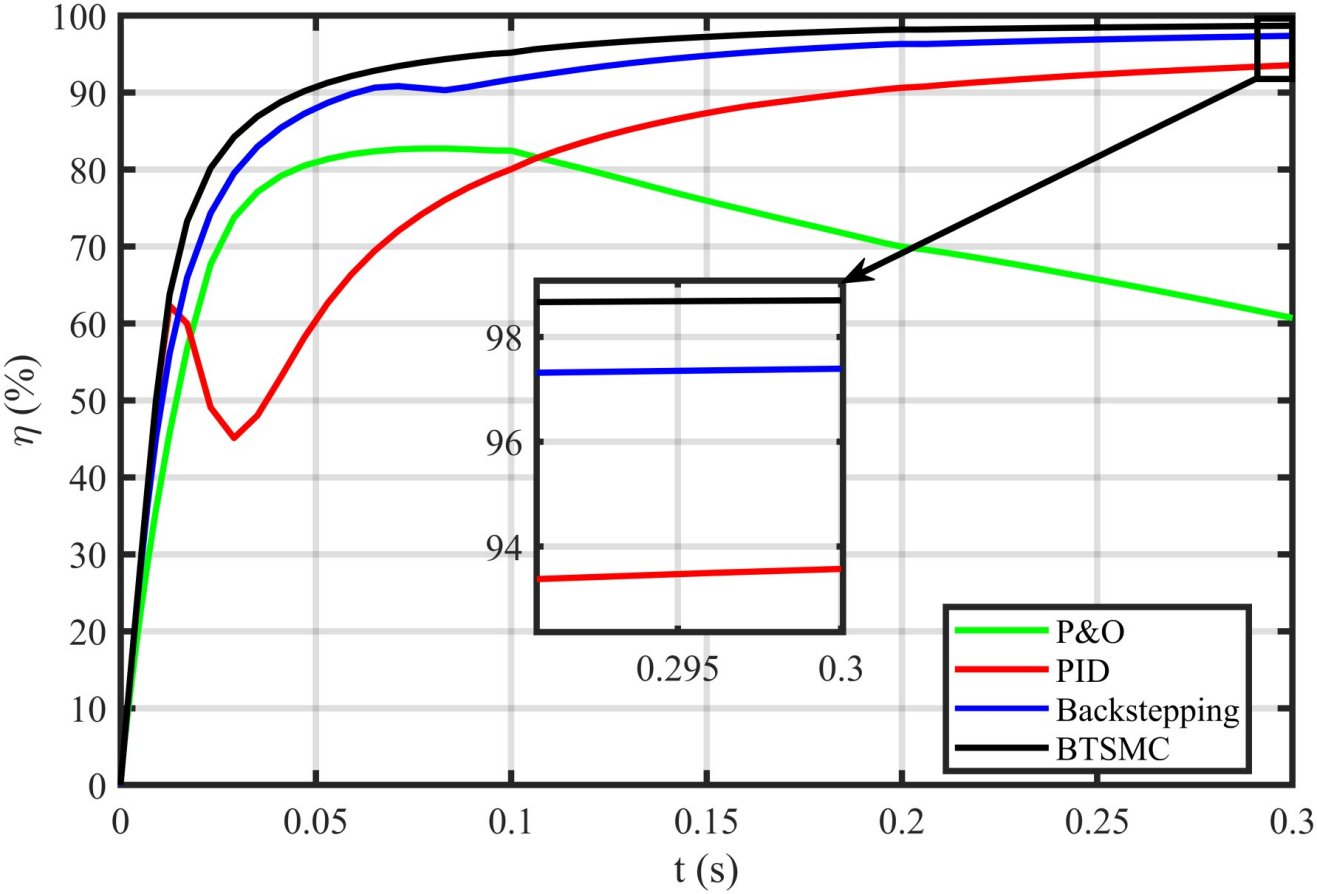
Efficiency (*η*) under varying climatic and faulty conditions.

In this case, multiple faults are injected in the plant under varying conditions of temperature, irradiance, and load. In this work, two uncertain conditions are introduced in the system. In the time interval 0.06 to 0.08 seconds, a fault, *x*_2*f*_ = 0.5*μ* sin(*t*)/*C*_*x*_, is inoculated in inductor current *x*_2_, Δ*x*_2_ = *x*_2_+*x*_2*f*_. In the time interval of 0.16 to 0.18 seconds, a uncertainty of Δ*C* = 0.48*nF* is introduced in output capacitor ‘*x*_3_’, as Δ*x*_3_ = *C*_*y*_ + Δ*C*. The output voltage of PV deviates from the reference, as depicted in Fig 16. The proposed controller shows robustness as compared to other existing techniques in faulty conditions. It can be observed that the BTSMC controller attains steady-state earliest than the other MPPT techniques. The PV output power is shown in Fig 17. It clearly shows that the proposed control technique outperforms its contenders in this scenario. The performance indices (IAE, ITAE, ISE, and ITSE) of this PV system under faulty conditions are presented in Figs 18–21. The effective performance of developed control technique is verified by the least value in these indices. The proposed BTSMC has transmitted the power to load with the efficiency of 98.72%, as shown in Fig 22. These results illustrate that the developed control technique outperforms the existing competitive MPPT techniques in this scenario of parametric variations. The proposed control technique is shown robustness against the faulty conditions and having less chattering effect, high rise time, better settling time, improved transient response, fast-tracking precision, and convergence. The statistical analysis of all MPPT techniques is presented in Table 3.

**Table 3:** Statistical analysis of MPPT techniques.

Measuring	Under varying climatic conditions				Under faulty condition			
Parameters	BTSMC	B	PID	P&O	BTSMC	B	PID	P&O
IAE	111	140	393	457	112	180	400	2200
ITAE	4.35	5.52	11.2	69	4.67	9	12	410
ISE	1.04	1.15	4.23	2.66	1.11	1.21	4.5	20
ITSE	0.43	0.44	0.76	2.44	0.44	0.65	0.79	40
*η* (%)	98.74	98.14	93.71	92.48	98.72	97.38	93	62

## 6 Conclusion and future work

The article presented the BTSMC control scheme for the MPPT application of PV systems. The buck-boost converter is used as an interface between load and PV array. The non-inverting topology of the buck-boost converter has been utilized in this work. To attain maximum power, the duty cycle of the converter is controlled through BTSMC. The reference of voltage has been generated by the RBF NN. The finite-time stability of the system has been verified using the Lyapunov stability function. The proposed controller performance is validated by comparison with P&O, PID, and backstepping controller under varying conditions of temperature, irradiance, and load. Furthermore, the developed control technique is again compared with its existing contenders under varying conditions of load and environment with fault and uncertainty. The proposed controller has been outperformed its existing competitors in all scenarios. The performance indices show the best performance of the proposed control technique in all conditions as compared to other existing techniques. Although, the proposed controller performs exceptionally well but it depends upon the reference values of the RBF NN plane. It is needed to update the RBF NN plane due to any type of failure in the PV system for optimal performance of the controller. In this manner, the generation of reference may demand the amalgamation of other algorithms like ACO and PSO to generate maximum power successfully.

The possible directions for future research work include;

- Comparison of proposed control schemes with other different MPPT techniques under partial shading conditions (PSC).
- Integration of the PV system with grid and parallel operation with other renewable energy systems.
- Application of the different inverter topologies to transfer the PV array output power into AC electric appliances.
- Development of the hybrid energy storage system to store energy for peak hours.
- Hardware-level implementation of proposed nonlinear control algorithms under varying environmental conditions.
